# Supplementary material for: The genome sequence of the Dune Robberfly, Philonicus albiceps (Meigen, 1820)
Source: Wellcome Open Res. 2025 Jul 14;10:349. [Version 1] doi: 10.12688/wellcomeopenres.24346.1 (PMC12756592; doi:10.12688/wellcomeopenres.24346.1)
Supplement: Peer review history

# Reviewer report: Reviewer response for version 1

**DOI:** 10.21956/wellcomeopenres.26847.r142124

**Published:** 2026-01-20

**Authors:** Arun Arumugaperumal

**Affiliations:** 1 Rajalakshmi Engineering College, Chennai, India

The authors have described the genome sequencing project of the Dune Robberfly, *Philonicus albiceps*. They have used a combination of PacBio long-read sequencing and Hi-C sequencing data to obtain a high-quality genome assembly. The assembly reported here is of size 253.92 Mb, spread among 6 chromosomes. A female specimen has been used for sequencing. But the sex chromosome was not identified. The mitochondrial genome of size 16.9 kb has also been assembled. The sequence data have been submitted to public databases. Annotations were obtained from the EMBL pipeline and 11,887 protein-coding genes have been reported. BUSCO analysis has shown that the sequences are 96.3% complete with respect to diptera_odb10 reference. The quality of the genome is evident from the number of genes predicted and the high N50 value. The links to datasets provided in the article are active.

 The figures are not visible in the online version of the article.

Are sufficient details of methods and materials provided to allow replication by others?

Yes

Is the rationale for creating the dataset(s) clearly described?

Yes

Are the datasets clearly presented in a useable and accessible format?

Yes

Are the protocols appropriate and is the work technically sound?

Yes

Reviewer Expertise:

Bioinformatics; Genomics

I confirm that I have read this submission and believe that I have an appropriate level of expertise to confirm that it is of an acceptable scientific standard.

# Reviewer report: Reviewer response for version 1

**DOI:** 10.21956/wellcomeopenres.26847.r142129

**Published:** 2025-12-31

**Authors:** Laurence Despres

**Affiliations:** 1 Laboratoire d'Ecologie Alpine (Ringgold ID: 56837), Grenoble, Auvergne-Rhône-Alpes, France

The authors report on the chromosome-level genome assembly of a female specimen of the Dune Robberfly *Philonicus albiceps* (Diptera; Asilidae) a common sand habitat specialist found on the coasts throughout the Palaearctic. The genome sequence has a total length of 253.92 megabases. The assembly contains two haplotypes with most of haplotype 1 scaffolded into 6 chromosomes. The mitochondrial genome has also been assembled.

 The quality of the assembly was further assessed by the proportion of complete BUSCO genes recovered, which is very high (96.6% of the expected gene set of the using the diptera_odb10 reference set). RNA sequencing data were also generated. The genome assembly is of good quality and was constructed using appropriate methods: Pac Bio HiFi long reads (91 X coverage) and Hi-C data to confirm scaffolding, following the high standard of the Darwin Tree of Life Project for results presentation.

Are sufficient details of methods and materials provided to allow replication by others?

Yes

Is the rationale for creating the dataset(s) clearly described?

Yes

Are the datasets clearly presented in a useable and accessible format?

Yes

Are the protocols appropriate and is the work technically sound?

Yes

Reviewer Expertise:

evolutionary ecology and genomics

I confirm that I have read this submission and believe that I have an appropriate level of expertise to confirm that it is of an acceptable scientific standard.

# Reviewer report: Reviewer response for version 1

**DOI:** 10.21956/wellcomeopenres.26847.r138634

**Published:** 2025-11-14

**Authors:** Darren Obbard

**Affiliations:** 1 The University of Edinburgh, Edinburgh, Scotland, UK

This data note reports the sequencing and assembly of the genome of Philonicus albicepsas part of the “Darwin Tree of Life” programme. In common with other data notes from this research effort, the reporting is standardized and quite brief. As such, I have very few comments to make.

 The approach is state-of-the-art, the raw data appear to be of a suitably high quality, and the assembly methods are appropriate. The public availability of raw data and genome assembly are appropriate. The resulting genome is likely to be of very high quality, and I have no doubt that it will be of great value to any researchers working on asilids and other diptera, or on the comparative or evolutionary genomics of insects more generally.

 The biological context and detail are very good, and much better than many of these genome reports.

 My minor suggestions are:

 (1) In addition to the sequenced specimen itself, it would be nice to have an ‘in life’ photograph and/or perhaps both sexes. It would be especially nice to have one in which the diagnostic white bristles were visible. CC-BY images seem to be available from iNaturalist. Given the specific habitat, and habitat view might also be a nice addition.  

 (2) Is Stubbs & Drake the intended reference for all of the statements in the paragraph beginning “ *Philonicus albiceps *has a Palaearctic distribution …”. The paragraph feels like it is lacking references, and this may be why – perhaps emphasize that all of the information is from a single source. Also, this paragraph contains a sentence beginning ‘P.’, i.e. starting with an abbreviation - which is generally considered bad practice

 (3) It is odd that the methods mention the individual was female, but final paragraph of the introduction does not.

Are sufficient details of methods and materials provided to allow replication by others?

Yes

Is the rationale for creating the dataset(s) clearly described?

Yes

Are the datasets clearly presented in a useable and accessible format?

Yes

Are the protocols appropriate and is the work technically sound?

Yes

Reviewer Expertise:

Evolutionary genetics and genomics of invertebrates and their pathogens

I confirm that I have read this submission and believe that I have an appropriate level of expertise to confirm that it is of an acceptable scientific standard.
